# Impact of *OXTR* Polymorphisms on Subjective Well-Being: The Intermediary Role of Attributional Style

**DOI:** 10.3389/fgene.2021.763628

**Published:** 2022-02-09

**Authors:** Lei Ji, Changfeng Chen, Binyin Hou, Decheng Ren, Fan Yuan, Liangjie Liu, Yan Bi, Zhenming Guo, Fengping Yang, Xi Wu, Fujun Chen, Xingwang Li, Chuanxin Liu, Zhen Zuo, Rong Zhang, Zhenghui Yi, Yifeng Xu, Lin He, Yi Shi, Tao Yu, Guang He

**Affiliations:** ^1^ Key Laboratory for the Genetics of Developmental and Neuropsychiatric Disorders, Ministry of Education, Bio-X Institutes, Shanghai Jiao Tong University, Shanghai, China; ^2^ Shanghai Key Laboratory of Psychotic Disorders, Shanghai Mental Health Center, Shanghai, China; ^3^ Jining Medical University, Jining, China; ^4^ Shanghai Center for Women and Children’s Health, Shanghai, China

**Keywords:** *OXTR* gene polymorphisms, subjective well-being, attributional style, intermediary role, rs2254298 SNP

## Abstract

Polymorphisms in the oxytocin receptor (*OXTR*) gene are related to individual differences in negative emotions, such as depressive symptoms and anxiety. However, it remains unclear what the potential roles of *OXTR* polymorphisms are in subjective well-being (SWB), which is negatively correlated with depressive symptoms. We examined attributional styles as mediator between SWB and five polymorphisms of the oxytocin receptor gene (*OXTR* rs53576, rs2254298, rs1042778, rs2268494, and rs2268490) among 627 full-time college freshmen (*M*_*age*_ = 20.90, *SD* = 0.82 for male; *M*_*age*_ = 20.81, *SD* = 0.92 for female) using structural equation modeling. The results showed that individuals with the *OXTR* rs2254298 AA genotype and rs53576 AA/GA genotype reported higher scores on SWB, which suggested that individuals with this genotype experienced more happiness. Moreover, external attributional style partially mediated the association between *OXTR* rs2254298 polymorphism and SWB (*β* = 0.019, 95%*CI* [0.001, 0.036], p = 0.035). In conclusion, our findings demonstrated that the genetic variations of *OXTR* played a role in the individual differences of SWB, and external attribution style could mediate the association.

## Introduction

Subjective well-being (SWB), popularly referred to as happiness or satisfaction (Diener, Oishi, & Tay, 2018), is defined as a person’s cognitive and affective evaluations of their life (Diener, 1984). SWB has three components: life satisfaction, positive affect, and negative affect (the three factors are independent and should be measured separately) (Diener et al., 2018; Myers and Diener, 1995). A person with a high level of SWB tends to report more life satisfaction, greater positive affect, and less negative affect (Myers and Diener, 1995); also, striving for happiness seems to be the leading life purpose. There is increasing evidence that SWB would be a cause of health and social relationships rather than a mere consequence, which implies that it is critical to college students’ psychological and physical development (Diener et al., 2017). SWB is reported to differ in multiple cultures, such as individualist cultures being happier than collectivists, which may be due to the diverse components and conception of SWB across different societies (Suh and Oishi, 2002; Diener et al., 2017). In addition, methodological challenges to assessing SWB contributed to the inconsistency (Diener et al., 2017). It was not sufficient for measuring SWB based solely on self-reported scales, and an integrated approach including three dimensions of SWB should be applied.

As a complex trait, SWB is influenced by many factors ranging from genetics to societal conditions (Anglim, Horwood, Smillie, Marrero, and Wood, 2020; Bartels, 2015; Litwin and Shiovitz-Ezra, 2011). Previously, most research explored the social, economic, and psychological impacts on SWB (Seligman and Csikszentmihalyi, 2000). There are relatively few studies in the area of genetic loci on SWB, despite the fact that SWB can be attributable to genetics. Meta-analyses have shown SWB has moderate heritability, ranging from 0.32 to 0.41 (Bartels, 2015; Bartels et al., 2010; Costa McCrae, 1980; De Neve, et al., 2012; Røysamb and Nes, 2018), which indicates that around 35% of individual variation in SWB is attributable to genetic effects. To date, several studies have attempted to explore the genetic polymorphisms associated with SWB. The transcription efficiency of serotonin transporter genes (5-*HTTLPR*) was reported to be positively related to life satisfaction among Americans (De Neve, 2011), but follow-up work on a newly independent replication sample did not replicate the significant finding (De Neve et al., 2012). Japanese individuals with the CC genotype of rs806377 (*CNR1*) had the highest level of subjective happiness, whereas Canadian individuals with the TT genotype of rs806377 had the highest level (Matsunaga et al., 2018). Authors attributed the inconsistencies to cultural differences in the concept of happiness. Among the Chinese, the TC genotype of rs454214 (*MFE2C*) was found to be positively associated with SWB (Hou et al., 2020), while rs7973260 (*RAPGEF6*), rs3756290 (*RAPGEF6*), and rs4481363 (*LOC105377703*) were not associated with SWB (Wang et al., 2017). However, genome-wide association studies (GWAS) conducted in the European population showed that rs3756290 was significantly associated with SWB, as were rs4958581 and rs2075677 (*CSE1L*) (Okbay et al., 2016). Population stratification should be considered appropriately in GWAS, as diverse ethnic groups and populations may cause spurious associations (Shigemizu et al., 2021). In addition, gene polymorphisms may be implicated in the gene-culture interaction. As suggested in previous literature, genetic susceptibility to complex traits should be examined in the context of environmental and social factors, which are inseparably linked to genetic susceptibility (Tam et al., 2019). Because of limited mobility, local adaptation, and the genetic drift, people from different cultures may have a different incidence of certain genotypes, leading to a false association between genotype and cultural attributes (Diener et al., 2018). It might explain why individuals with the same susceptibility genes may sometimes show different and even opposite phenotypes in different populations.

Recent studies have found that SWB is the outcome of the interaction between various endogenic and exogenic factors (Krol, Kamboj, Curran, and Grossmann, 2014). Oxytocin (*OXT*) is one of the critical endogenic mediators and is increasingly recognized as a key regulator in many complex social behaviors and mental disorders. It contributes to the inhibition of the stress-induced hypothalamic-pituitary-adrenal (HPA) axis, anti-depressive effects, enhancement of well-being, and promotion of social behavior (Scantamburlo et al., 2007; Ishak, Kahloon, and Fakhry, 2011). Furthermore, *OXT* could facilitate positive social support, trust and social interactions (Kosfeld, Heinrichs, Zak, Fischbacher, and Fehr, 2005), which are strongly associated with SWB. Oxytocin receptor (*OXTR*) mapping to 3p25 has four exons and three introns, and the *OXTR* gene is the main target for endogenous and synthetic *OXT*. Human studies particularly emphasized the involvement of two single nucleotide polymorphisms (SNP, rs53576, rs2254298) in *OXTR*, which is the focus of our research (Feldman, Monakhov, Pratt, and Ebstein, 2016).

The SNP rs53576 appears to be a promising candidate to explain the differences in positive affect. Lucht et al. investigated the associations among *OXTR* polymorphisms, positive and negative affect, social and emotional loneliness, and intelligence in normal German subjects, and found that men with the AA genotype of *OXTR* rs53576 showed lower positive affect scores than those with other genotypes (Lucht et al., 2009). This view was supported by Saphire-Bernstein et al. who demonstrated that *OXTR* rs53576 A-allele carriers showed lower optimism and higher self-reported depression among 326 students and employees of a large Western university (Saphire-Bernstein, Way, Kim, Sherman, and Taylor, 2011). Conversely, Costa et al. found that the GG genotype of *OXTR* rs53576 is positively associated with depression in a clinical sample (Costa et al., 2009), and Kushner suggested that GG homozygotes increase susceptibility to depressive symptoms among early adolescent youth (Kushner, Herzhoff, Vrshek-Schallhorn, and Tackett, 2018). Costa et al. also identified an association between the GG homozygosity of the rs2254298 and depression. The positive correlation between GG genotype of rs2254298 and depression was also observed in the Caucasian sample (Thompson, Parker, Hallmayer, Waugh, and Gotlib, 2011). For the other three OXTR polymorphisms, the research to date has tended to focus on interpersonal relationships rather than emotions. The TT genotype of the *OXTR* rs1042778 (located in the 3′ UTR) was reported to be associated with less empathic communication (Schneiderman, Kanat-Maymon, Ebstein, & Feldman, 2014). The CC/TT in rs2268490 had protective effects for general trust by buffering job stress in healthy Chinese university teachers (Fang et al., 2020). Additionally, the *OXTR* SNPs rs2268494 and rs2268490 were combined into a cumulative genetic risk index which could predict observed empathy during support-giving interactions in new lovers (Schneiderman et al., 2014). Together, these studies indicate that rs53576 and rs2254298 may be linked with individual differences in SWB.

Attributional style may contribute to individual differences in subjective well-being. Attributional (or explanatory) style is a personal cognitive style reflecting a standard specific way of explaining the reasons of events in which people are involved (Heider, 1958). It involves three dimensions that influence how we explain an outcome: internality versus externality, stability versus instability, and globality versus specificity (Peterson, 1991). We focused on the internal-external dimension that psychologists are generally concerned with. The difference between internality and externality is whether they tend to attribute events to the self or to other factors. Prior literature recommended that attributional style was moderately influenced by genetic effects, and the association between attributional style and depression also reflected considerable genetic effects (Lau, Rijsdijk, and Eley, 2006). Most literature has linked attributional style to depressive symptoms (Sweeney, Anderson, and Bailey, 1986; Tianqiang, Zhang, Dajun, Yang, and Zhengzhong, 2015), which in turn relates to SWB. Researchers found that attributions to internal, stable, and global causes were significantly associated with depression for negative events, while attributions to external, unstable, and specific causes were significantly associated with depression for positive events (Sweeney et al., 1986). Specifically, ability and luck attribution factors were associated with depression according to this study (Sweeney et al., 1986). In terms of positive affect, O'Donnell showed that SWB might partially depend on a sense of control and a positive explanatory style for events in college students (O'Donnell, Chang, and Miller, 2013). In addition, people could upregulate happiness through internal attributions (vs. external) by recruiting the parahippocampal gyrus (Loeffler et al., 2018).

In light of the possibility that genetic influences play a role in the formation of attributional style, the implications for models of SWB are interesting. The genetic basis of the relationship between attributional styles and SWB has received little attention. Although there is now evidence that genetic factors contribute to SWB, it is unclear how these genetic factors are expressed through psychosocial pathways to affect individual differences in SWB. One possibility is that cognitive factors, such as attributional style, contribute to some genetic variants associated with SWB. As a result, we hypothesized that the *OXTR* SNPs rs53576 and rs2254298 were the genetic locus of SWB, and that people who were more genetically predisposed toward specific attributional styles had corresponding levels of SWB. Given the inconsistency of findings on the genetic variants of SWB in different populations, our study may aid in understanding the relationship between attributional styles and SWB among Chinese college students from a genetic perspective.

## Materials and Methods

### Participants

During the first month of college enrollment, 627 full-time college freshmen (63.64% female, mean age = 20.84 ± 0.89 years) were randomly recruited from Jining Medical University, Jining, China. All of these college freshmen signed the informed consents and this study was appraised and approved by the Ethics Committee of Jining Medical University and Shanghai human genetic resources (JNMC-2016-KY-001). All methods were carried out following the relevant guidelines and regulations. The questionnaires include demographic features, assessment of SWB, and interpersonal attributional style as well as survey feedback (efficacy, understanding, carefulness, significance).

### Measurements

#### Subjective Well-Being

As we stated earlier, SWB has been widely conceptualized as life satisfaction, the presence of positive affect, and the relative absence of negative affect (Myers and Diener, 1995). Thus, life satisfaction, positive affect, and negative affect were used as indicators of the latent variable of SWB in this study. Life satisfaction was assessed with the Satisfaction With Life Scale (SWLS) (Diener, Emmons, Larsen, and Griffin, 1985), and positive affect and negative affect were measured with the Positive Affect (PA) and Negative Affect (NA) subscales from the Positive and Negative Affect Scale (PANAS) (Watson, Clark, and Tellegen, 1988) (see more details in Supplementary Appendix SA,B and Supplementary Material S1).

SWB was modeled as a single factor indicated by the SWL and PANAS subscales (Goodman, Disabato, Kashdan, and Kauffman, 2018). We conducted confirmatory factor analyses for the final score of SWB and had acceptable model fit [*χ* ^2^ (3) = 426.475, p < 0.001] (Bentler, 1980). The loadings of the measured variables on the latent variable of SWB were statistically significant at the 0.001 level, which implied that SWB had been adequately measured by its respective indicators.

#### The Multidimensional–Multiattributional Causality Scale (MMCS)

We used MMCS to assess the attributional style in this study (Lefcourt, Von Baeyer, Ware, and Cox, 1979). The MMCS is a self-reported scale consisting of two scales for measuring achievement locus of control scale (24 items) and affiliation locus of control scale (24 items) (Supplementary Appendix SC). Oxytocin is mainly related to interpersonal affiliation; therefore, the present study is concerned only with the affiliation attributional scale. The affiliation MMCS consists of four 6-item subscales designed to measure ability, effort, context, and luck attribution, ranging from 0 (disagree) to 4 (agree) (e.g., “Making friends is fun and sometimes I have to attribute it to luck”; “It takes effort to maintain friendships”) (Lefcourt et al., 1979). Moreover, subscales could be summed to obtain measures of internality (ability and effort) and externality (context and luck) (Lefcourt, 1981). Previous research reported the alpha coefficient for the affiliation scale ranged from 0.70 to 0.84 and had good construct validity (Lefcourt et al., 1979). The alpha coefficient in this study is 0.86.

### SNP Selection and Genotyping

Five SNPs in the *OXTR* (rs53576, rs2254298, rs1042778, rs2268494, and rs2268490) gene were selected for inclusion in this study, which was particularly underscored in human research (Feldman et al., 2016). More detailed information about the SNPs is presented in Table 1.

**TABLE 1:** *OXTR* single nucleotide polymorphism characteristics

SNP	Chromosome location	Molecular consequence	Major/minor allele	MAF^*^	Genotype frequencies N (%)			HWE p
rs53576	chr3: 8762685	intron_variant	A/G	0.33	AA: 301 (48.2)	AG: 263 (42.1)	GG: 60 (9.6)	0.945
rs2254298	chr3: 8760542	intron_variant	G/A	0.15	GG: 291 (46.4)	GA: 276 (44.0)	AA: 60 (9.6)	0.896
rs1042778	chr3: 8752859	3_prime_UTR_variant	G/T	0.40	GG: 529 (84.5)	GT: 92 (14.7)	TT: 5 (0.8)	0.899
rs2268494	chr3: 8760360	intron_variant	T/A	0.07	TT: 544 (87.2)	TA: 78 (12.5)	AA: 2 (0.3)	0.876
rs2268490	chr3: 8755399	intron_variant	C/T	0.15	CC: 171 (27.4)	CT: 308 (49.3)	TT: 146 (23.4)	0.937

SNP, single nucleotide polymorphism; MAF^*^, minor allele frequency observed in our sample; HWE, Hardy–Weinberg equilibrium; *OXT*, oxytocin gene; chr, chromosome.

DNA was extracted from the peripheral venous blood of each freshman using the Trizol protocol. Genotyping was conducted by the matrix-assisted laser desorption/ionization time of flight (MALDI-TOF) mass spectrometer on the MassARRAY® Analyzer 4 platform (Sequenom, San Diego, CA, USA). My-Sequenom online software Assay Design Suite v2.0 was applied to design probes and primers (Supplementary Material S2). For the genetic analyses, we employed SHEsisPlus (http://shesisplus.bio-x.cn/SHEsis.html) to analyze allelic and genotypic distributions and the Hardy–Weinberg equilibrium (Shen et al., 2016). In this sample, the distribution of both genotypes showed no deviation from the Hardy–Weinberg equilibrium.

### Statistical Analysis

To reach the minimal regression coefficient of determination *r* ^2^ (0.010–0.015), a sample of 592–892 participants was required (two-tailed *α* = 0.05, 1-*β* = 0.85) based on the power analysis conducted with G*Power 3.0. The post hoc power analysis indicated that our sample size was appropriate. For all analyses, we set statistical significance at p < 0.05. First, descriptive statistics were calculated for all study variables, with an additional *t*-test for different genders. Second, correlation analysis and its heat map between SWB and attributional style were obtained employing the “corrgram” package. Third, the association between each SNP with SWB and attributional styles in five genetic models (codominant, dominant, recessive, over-dominant, and log-additive models, respectively) was acquired with the “SNPassoc” R package, respectively (Gonzalez et al., 2007). To verify which attributional style was associated with the certain genotype, the linear regression analysis with SWB relevant genotype as predictor was conducted. The false discovery rate (FDR) was applied to our linear regression analysis to reduce the possibility of false positives. Only those variables that are significant in both regression analysis and genetic association analysis will be considered for the subsequent mediation analysis. Finally, multiple mediator analyses were conducted to estimate the total, direct, and indirect effects of the predictor (rs2254298) on the outcome (SWB) via two mediators (context attribution and luck attribution) controlling for gender by “lavaan” 0.6–7 package, which is based on maximum likelihood estimations (Preacher & Hayes, 2008; Rosseel, 2012). *OXTR* rs2254298 was coded as dummy variables (AA = Yes, GA + GG = No) in the structural equation modeling (SEM). chi-Square values, root mean square error of approximation (RMSEA), comparative fit index (CFI), and Tucker–Lewis index (TLI) were used as measures of fit. The model fit is considered to be accepted by convention if the RMSEA is less than or equal to 0.08, and the CFI and TLI should be equal to or greater than 0.90 (Hu and Bentler, 1999). To assess the significance of mediation effects, we used a recommended procedure and calculated the 95% confidence intervals of 1,000 bias-corrected and accelerated bootstrapping analyses (Mackinnon, Lockwood, & Williams, 2004; Hayes, 2013).

### Transparency and Openness

We report how we determined our sample size, all manipulations, and all measures in the study, and we follow JARS (Kazak, 2018). The data presented in this study can be found in online repositories, and the code is available on request from the corresponding author. Data were analyzed using R studio (version 4.0.2). Supplementary Material S2 summarizes the R packages we used in this study. This study’s design and its analysis were not pre-registered.

## Result

### Descriptive statistics and correlations between main study variables

**FIGURE 1:**
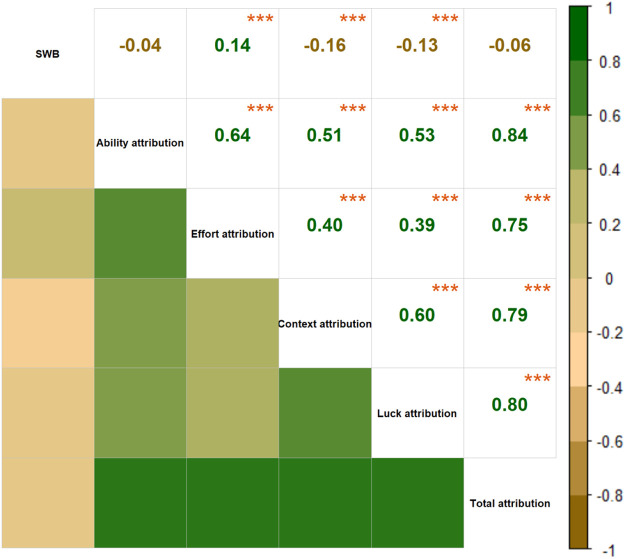
Heat map illustrating correlations between subjective well-being and attributional style. The values in the upper triangular matrix are correlation coefficients. In the lower triangular matrix, the darker the color is, the stronger correlation is. Positive correlations are shown in green, while negative correlations are shown in brown. Except for the correlation between subjective well-being and ability attribution and total attribution, the other correlation coefficients were significant at 0.001 level. *p < 0.05; **p < 0.01; ***p < 0.001.

Descriptive statistics and gender comparisons were presented in Table 2. Gender comparisons revealed higher levels of affiliation attributional style in ability, effort, and luck among females. Figure 1 presents the results obtained from the correlational analysis for SWB and the four dimensions of attributional style. The results showed that SWB was positively correlated to effort attribution (*r* = 0.14, p < 0.001), and negatively correlated to context (*r* = −0.16, p < 0.001) and luck attribution (*r* = −0.13, p < 0.001).

**TABLE 2:** Characteristics of the study population

	Range	Total	Male (N = 227)	Female (N = 400)	p
Age	18, 24	20.84 (0.89)	20.90 (0.82)	20.81 (0.92)	0.244
Residence = Urban (%)		229 (36.5%)	92 (40.5%)	137 (34.2%)	0.138
Minority = Han (%)		607 (96.8%)	218 (96.0%)	389 (97.2%)	0.552
Family history of psychosis = Yes (%)		39 (6.2%)	9 (4.0%)	30 (7.5%)	0.112
SWB (mean (SD))	-2.4, -2.5	0 (0.92)	0.02 (0.92)	-0.01 (0.91)	0.647
SWL	5, 35	20.35 (5.89)	20.50 (6.06)	20.26 (5.80)	0.629
PANAS.pos	16, 48	32.58 (5.57)	32.85 (5.53)	32.42 (5.59)	0.356
PANAS.neg	10, 47	24.63 (7.07)	25.12 (7.09)	24.34 (7.04)	0.188
MMCS (mean (SD))					
Ability attribution	4, 24	13.26 (3.33)	13.62 (3.42)	13.06 (3.27)	**0.045**
Effort attribution	3, 24	14.28 (3.28)	14.70 (3.23)	14.04 (3.28)	**0.015**
Context attribution	5, 24	14.04 (3.28)	14.13 (3.29)	14.00 (3.28)	0.633
Luck attribution	0, 24	12.24 (3.66)	12.70 (3.50)	11.97 (3.72)	**0.017**

SWL: the satisfaction with life scale.

PANAS: the positive and negative affect scale.

MMCS: The Multidimensional–Multiattributional Causality Scale.

Bold values indicate significant level (*p* < 0.05).

### Association Analysis

The results of association analysis using genetic models were presented in Supplementary 2. Two *OXTR* polymorphisms, rs53576 and rs2254298, were significantly associated with SWB fitting the recessive models. The GA + AA genotype of *OXTR* rs53576 polymorphism (p = 0.004, corrected p = 0.020), and AA genotype of rs2254298 (p = 0.014, corrected p = 0.035) were significantly related to higher level of SWB. We thus used rs53576 and rs2254298 as predictors of linear regression to examine their associations with four dimensions of attributional style. As shown in Table 3, the genotype of *OXTR* rs53576 could not predict attributional styles and thus was not considered in the subsequent mediation analysis. For rs2254298, the AA genotype could predict the lower level of ability attribution (p = 0.028, corrected p = 0.036), context attribution (p = 0.029, corrected p = 0.036), and luck attribution (p = 0.004, corrected p = 0.020). The association analysis using genetic models provided the same results, and detailed statistics are shown in Supplementary Material S2. Based on the recessive model, AA genotype of *OXTR* rs2254298 was significantly associated with lower score of ability attribution style (p = 0.0181), context attribution style (p = 0.0291) and luck attribution style (p = 0.0041). These results in the preliminary analysis indicated that *OXTR* rs2254298 was correlated to SWB and three dimensions of attributional styles. The next analysis, therefore, moved on to examine whether the three dimensions of attributional styles could mediate the association between *OXTR* rs2254298 and SWB.

**TABLE 3:** Regression analyses on the impacts of *OXTR* SNPs on affiliation attributional styles

Variable	rs53576		Statistic of regression analyses					rs2254298		Statistic of regression analyses				
	GG	GA + AA	*β*	*R* ^2^	t	p	fdr	GA + GG	AA	*β*	*R* ^2^	t	p	fdr
SWB	−0.32 (0.91)	0.03 (0.91)	−0.36	0.013	−2.88	**0.004**	**0.02**	-0.03 (0.91)	0.28 (0.96)	0.306	0.01	2.475	**0.014**	0.035
Ability attribution	0.27 (1.01)	0.04 (0.99)	0.231	0.005	1.721	0.086	0.173	0.09 (1.00)	−0.21 (0.90)	−0.3	0.008	−2.2	**0.028**	0.036
Effort attribution	0.16 (0.97)	0.05 (0.97)	0.108	0.001	0.827	0.409	0.409	0.08 (0.97)	−0.07 (0.94)	−0.15	0.002	−1.16	0.246	0.246
Context attribution	0.15 (1.13)	-0.04 (0.99)	0.193	0.003	1.422	0.156	0.195	0.01 (1.02)	−0.29 (0.83)	−0.3	0.008	−2.19	**0.029**	0.036
Luck attribution	0.26 (0.97)	0.04 (0.99)	0.22	0.004	1.63	0.104	0.173	0.10 (0.99)	−0.29 (0.93)	−0.39	0.013	−2.88	**0.004**	0.020

Bold values indicate significant level (*p* < 0.05).

### Mediation analysis

**FIGURE 2:**
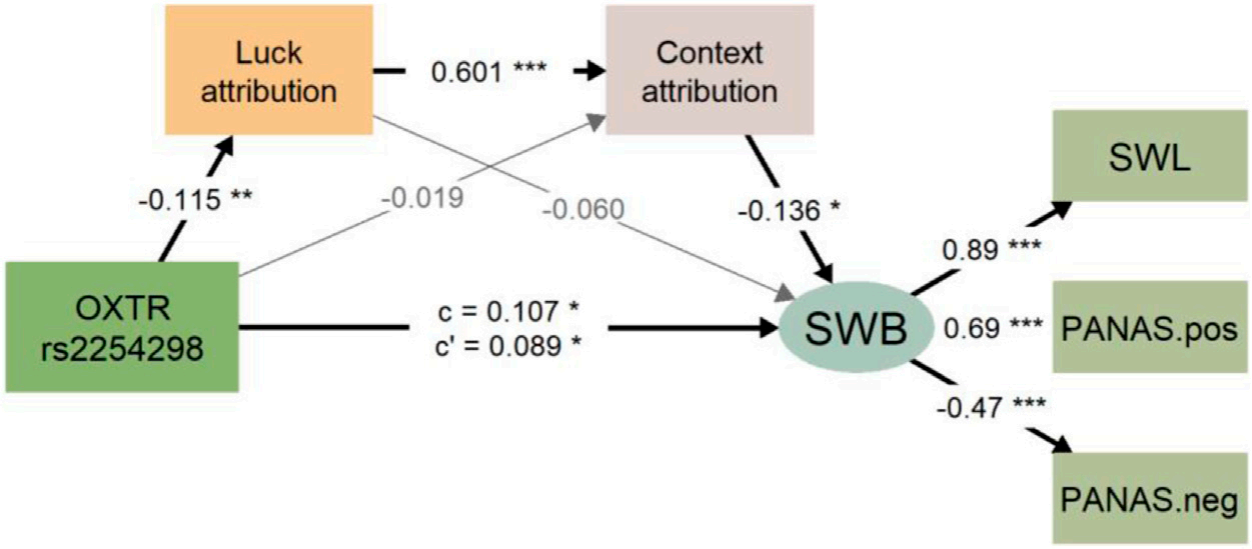
The figure shows the multiple mediations of context attribution style and luck attribution style in the association between *OXTR* rs2254298 and subjective well-being (SWB is a latent variable). All estimates are standardized, with * indicating p < 0.05, ** indicating p < 0.01, *** indicating p < 0.001, and grey line indicating nonsignificant. Note: “c” refers to the total effect of *OXTR* rs2254298 on subjective well-being; “c’” refers to the direct effect of *OXTR* rs2254298 on subjective well-being after controlling for context attribution and luck attribution.

Structural models were used to test the mediating role of attributional styles in the relationship between *OXTR* rs2254298 and SWB after controlling for the effect of gender (Figure 2). The final model showed a good model fit: *χ* ^2^_(8)_ = 39.034, RMSEA = 0.07, CFI = 0.959, TLI = 0.924, with bootstrapping the mediating effect 1,000 times. Table 4 presented the standardized path coefficients for the effects of *OXTR* rs2254298 on SWB in the mediation model. When both genotype and the context/luck attribution were included as predictors, the direct effect of rs2254298 genotype on SWB was still significant (*β′* = 0.089, p = 0.042, 95% CI 0.003, 0.174), accounting for 82.24% of the total effect (*β′* = 0.107, p = 0.018, 95%CI 0.019, 0.196). We found that the total indirect effect was 0.019, which accounted for 17.76% of the total effect in the relationship between the genotype of *OXTR* rs2254298 and SWB. The 95% confidence interval is 0.001–0.036. Specifically, the total indirect effect included three different pathways. *OXTR* rs2254298 affected students’ SWB partly through the mediating role of luck attribution, through the mediating role of context attribution, and through the chain mediating role of both luck attribution and context attribution, which were shown in indirect effects 1, 2 and 3, respectively. Furthermore, indirect effects 1, 2, and 3 accounted for 6.5%, 2.8%, and 8.4% of total effect, respectively, and the 95% confidence intervals overlap with zero, which indicated that indirect effect alone was not significant.

**TABLE 4:** Multiple mediation model test with bootstrapping

Mediators	Estimate	Se	z-value	95%*CI*	p Value
Direct Effect	0.089	0.044	2.032	(0.003, 0.174)	**0.042**
Indirect Effect 1 _(O→L→S)_	0.007	0.008	0.890	(−0.008, 0.022)	0.373
Indirect Effect 2 _(O→C→S)_	0.003	0.004	0.690	(−0.005, 0.010)	0.490
Indirect Effect 3 _(O→L→C→S)_	0.009	0.005	1.934	(0.000, 0.019)	0.053
Total Indirect Effect	0.019	0.009	2.111	(0.001, 0.036)	**0.035**
Total Effect	0.107	0.045	2.372	(0.019, 0.196)	**0.018**

Note. 95%CI: 95% Confidence Interval.

Bold values indicate significant level (*p* < 0.05).

Finally, to support our conceptual model, we compared it to three alternatives (Supplementary Material S1). The first (Supplementary Figure S1) measured ability attribution, luck attribution, and context attribution in a parallel model. This model showed less adequate fit to the data: *χ* ^2^_(11)_ = 570.696, TLI = −0.048, CFI = 0.451, RMSEA = 0.285. The second alternative model (Supplementary Figure S2) measured luck attribution and context attribution in a parallel model. This model also showed inadequate fit: *χ* ^2^_(7)_ = 314.32, TLI = 0.135, CFI = 0.596, RMSEA = 0.265, with all mediation paths insignificant. The third alternative model (Supplementary Figure S3) transposes luck attribution and context attribution. This model provided less adequate fit: χ^2^_(6)_ = 37.515, TLI = 0.897, CFI = 0.959, RMSEA = 0.092.

## Discussion

This study investigated the association between five *OXTR* polymorphisms: rs53576, rs2254298, rs1042778, rs2268494 and rs2268490, and individual differences in SWB and attributional styles. The findings suggested that variations at the *OXTR* rs53576 and rs2254598 loci were linked to SWB in Chinese college students. Individuals with the homozygote AA genotype of rs2254298 had higher SWB scores and lower scores on ability, luck, and context attributional styles than those with the GG/AG genotype. According to an SEM model, rs2254298 was directly associated with SWB, and the association was mediated by the chain mediating role of external attributional styles: from luck attribution to context attribution.

The potential role of *OXTR* gene polymorphism in depression and anxiety has been suggested by several studies, with little attention on positive affect. Given the strong genetic correlations between depressive symptoms and SWB (Okbay et al., 2016), this research examined the role of *OXTR* gene polymorphism on SWB in the context of Chinese cultures among college students. In both the recessive genetic models and regression analysis, the two *OXTR* SNPs, rs53576 and rs2254298, were significantly associated with SWB. Accordingly, it is reasonable to conjecture that the *OXTR* gene may be a genetic locus for SWB. As the result mentioned, individuals with the GG genotype of *OXTR* rs53576 polymorphism have a relatively lower degree of SWB in Chinese college students. This outcome reflects those of Costa et al. (2009) and Kushner et al. (2018) who also found a correlation between the GG genotype and depression, but is contrary to that of Lucht et al. (2009) and Saphire-Bernstein et al. (2011). These inconsistencies might be attributed to gene × culture interactions on SWB. Several studies revealed that the proportion of people with the rs53576 GG genotype was low in China (GG: 7.2%, AG: 42.3%, AA: 50.5%) (Luo et al., 2015; Butovskaya et al., 2016). In contrast, the studies of subjects from several western countries reported much higher frequencies of the rs53576 GG genotype (GG: 40.9%, AG: 45.9%, AA: 13.2%). The present study confirmed the previously published in Chinese (Luo et al., 2015). *OXTR* rs53576 has been found to interact with a cultural orientation to shape human empathy and the underlying neural correlates, suggesting that the G allele of *OXTR* rs53576 confers enhanced sensitivity to cultural norms (Kim et al., 2010; Luo et al., 2015). Thus, the ethnogeographic differences in the *OXTR* rs53576 genotype frequencies may be closely related to cultural differences in SWB.

The research of rs2254298 polymorphisms was mostly related to disorders like depression or autism. It was reported that the GG genotype of *OXTR* rs2254298 was positively linked to depression and anxiety in the Caucasian sample (Costa et al., 2009; Thompson et al., 2011), which was similar to our finding: GG individuals had lower SWB. The results suggest that the GG genotype carriers were less responsive to environmental contingencies, based on Brüne’s suggestion that the A allele of *OXTR* rs2254298 is a relatively recently evolved allele that often plays a role in depression (Brune, 2012). Besides, genetic variation in *OXTR* caused structural alterations in brain regions which may be involved in emotional regulation and social behaviors (Na et al., 2018). The anatomical studies have shown that AA genotype carriers of rs2254298 have greater amygdala volumes bilaterally than those with G carriers, which was replicated in three independent samples (Inoue et al., 2010; Furman, Chen, and Gotlib, 2011; Marusak et al., 2015). It seems plausible that greater amygdala volumes may be a protective factor for subjective mood. Our result complements previous findings and provides further evidence that *OXTR* SNPs are associated with positive outcomes (SWB) in young adults, promoting the further study of the hormone oxytocin in emotions and identification of the genetic mechanism of SWB.

Our study also suggested that high context attribution scores, high luck attribution scores, and low ability attribution scores were correlated with low levels of SWB. These results seem to be consistent with other research which found that individuals who regarded the causes of negative events as internal were more likely to develop future depression, while those who regarded the causes of positive events as external were more likely to have depression (Sweeney et al., 1986). Another interesting finding was that the AA genotype of *OXTR* rs2254298 appeared to be linked to attributional styles. We then explored a mediating effect of attributional styles on the relationship between *OXTR* SNP rs2254298 and subjective well-being. To the best of our knowledge, this is the first study of joint associations of *OXTR* variants and SWB with attributional styles. Based on the structural equation model, context and luck attribution rather than ability attribution did play a mediating role in the relationship between *OXTR* rs2254298 and SWB in our sample. The result also accords with earlier observations, which showed that the externality of attribution was related to depression (Banks and Goggin, 1983). Specifically, we found a chain-mediated model with two dimensions of external attribution, in which luck attribution is the cause of context attribution. College students with a high level of external attributional scores believe that their success lies not in their diligence and ability, but in more favorable situational conditions at the time, which would weaken their experience of SWB. This paper supports the involvement of the oxytocinergic system in the mechanisms that underlie SWB and specific attributional styles. *OXTR* rs2254298 is directly related to SWB (account for 82.2% in our model), and the genetic variants also improved SWB through the more optimistic external attribution. By further demonstrating the role of *OXTR* rs2254298 in individual differences in attributional styles, the findings have important implications for the protection of college students’ mental and physical health. It is necessary to cultivate college students’ better attribution style, reasonably recognize their psychological pressure, and improve the level of their SWB, especially for students with specific genotypes (Zhou and Hu, 2020). Otherwise, long-term irrational attribution will easily lead to self-resentment, regret, and other negative emotions. Certainly, the influences of SWB are by no means limited to these two determinants. Similar to depressive symptoms, it is a multifactorial, complex, and heterogeneous phenotype, and we will continue to explore other factors in future studies.

There are several limitations in this work that must be noted. First, the current sample for examining effects of genetic variants was relatively small compared to GWAS, which might limit the ability to detect smaller effects. It was suggested that GWAS signals might be the result of cryptic population stratification (Shigemizu et al., 2021), and the specific cultural trait plays a key role in interactions with genetic factors (Luo et al., 2015). Though our study adds to the understanding of SWB in Chinese cultural backgrounds, it is essential to replicate it in a larger and more diverse population to improve data accuracy through validation. Second, it is well recognized that complex traits and associated behavior are influenced by a large body of genetic variations and further affected by the environment (Litwin and Shiovitz-Ezra, 2011; Bartels, 2015; Anglim et al., 2020). It may provide further insight in the role of *OXTR* in SWB. Thus, more related factors, such as financial situation, need to be investigated as an important extension to acquire a more comprehensive understanding of the SWB in college students. Furthermore, SWB and attributional styles were not highly correlated in this study. Because SWB is strongly related to optimistic attributional styles (Cheng and Furnham, 2001), optimistic attributional styles with the Attributional Style Questionnaire, instead of the Multidimensional-Multiattributional Causality Scale, may be more applicable (Peterson et al., 1982). Third, due to lack of funds, only five most frequent SNPs were selected for the association study, so more SNPs in the *OXTR* gene are needed to be included as controls to repeat the results of this study on the basis of expanding the sample size. Furthermore, we could not conclude whether the *OXTR* genotype was the principal component in the association between gene polymorphisms and the SWB because we did not provide a replication sample. Forth, longitudinal research on SWB needs to be adopted for tracking the change of attribution styles and SWB during different periods to verify whether this result is still applicable. It would be helpful to further investigate causality and determine if certain correlates are indeed factors that contribute to SWB. Additionally, multiple methods, such as the Day Reconstruction Method [which can reduce memory biases inherent in the recall of feelings (Kahneman, Krueger, Schkade, Schwarz, & Stone, 2004)], can be integrated as beneficial supplements in future research.

## Conclusion

This study suggests that AA genotype of *OXTR* rs2254298 may predict individual differences in external attributional styles and subjective well-being. Furthermore, external attribution style appears to mediate the association between *OXTR* rs2254298 polymorphism and subjective well-being.

## Data Availability

The data has been uploaded to EVA repository Project: PRJEB49407 Analyses: ERZ4414822 https://www.ebi.ac.uk/eva/?eva-study=PRJEB49407.
