# Supplementary material for: Impact of OXTR Polymorphisms on Subjective Well-Being: The Intermediary Role of Attributional Style
Source: Front Genet. 2022 Feb 9;12:763628. doi: 10.3389/fgene.2021.763628 (PMC8864163; doi:10.3389/fgene.2021.763628)
Supplement: Supplementary file 2 [file DataSheet2.docx]

**Appendix a**

**The Satisfaction With Life Scale (SWLS) (Diener, Emmons, Larsen, & Griffin, 1985)**

You may agree or disagree with the following items. Please select the appropriate score to indicate how much you agree with each item below

|  | strongly disagree | disagree | somewhat disagree | neither agree nor disagree | somewhat agree | agree | strongly agree |
| --- | --- | --- | --- | --- | --- | --- | --- |
| 1. In most ways my life is close to my ideal | 1 | 2 | 3 | 4 | 5 | 6 | 7 |
| 1. The conditions of my life are excellent | 1 | 2 | 3 | 4 | 5 | 6 | 7 |
| 1. I am satisfied with my life | 1 | 2 | 3 | 4 | 5 | 6 | 7 |
| 1. So far I have gotten the important things I want inlife | 1 | 2 | 3 | 4 | 5 | 6 | 7 |
| 5. If I could live my life over, I would change almost nothing | 1 | 2 | 3 | 4 | 5 | 6 | 7 |

**Appendix b**

**The Positive and Negative Affect Scale (PANAS) (Watson, Clark, & Tellegen, 1988)**

This scale consists of a number of words that describe different feelings and emotions. Read each item and then list the number from the scale below next to each word. Indicate to what extent you feel this way right now, that is, at the present moment OR indicate the extent you have felt this way over the past week (circle the instructions you followed when taking this measure)

|  | Very Slightly or Not at All | A little | Moderately | Quite a bit | Extremely |
| --- | --- | --- | --- | --- | --- |
| interested | 1 | 2 | 3 | 4 | 5 |
| distressed | 1 | 2 | 3 | 4 | 5 |
| excited | 1 | 2 | 3 | 4 | 5 |
| upset | 1 | 2 | 3 | 4 | 5 |
| Strong | 1 | 2 | 3 | 4 | 5 |
| guilty | 1 | 2 | 3 | 4 | 5 |
| scared | 1 | 2 | 3 | 4 | 5 |
| hostile | 1 | 2 | 3 | 4 | 5 |
| enthusiastic | 1 | 2 | 3 | 4 | 5 |
| proud | 1 | 2 | 3 | 4 | 5 |
| irritable | 1 | 2 | 3 | 4 | 5 |
| alert | 1 | 2 | 3 | 4 | 5 |
| ashamed | 1 | 2 | 3 | 4 | 5 |
| inspired | 1 | 2 | 3 | 4 | 5 |
| nervous | 1 | 2 | 3 | 4 | 5 |
| determined | 1 | 2 | 3 | 4 | 5 |
| attentive | 1 | 2 | 3 | 4 | 5 |
| jittery | 1 | 2 | 3 | 4 | 5 |
| active | 1 | 2 | 3 | 4 | 5 |
| afraid | 1 | 2 | 3 | 4 | 5 |

**Appendix c**

**The Multidimensional-Multiattributional Causality Scale (MMCS) (Lefcourt, Von Baeyer, Ware, & Cox, 1979)**

You may agree or disagree with the following items. Please select the appropriate score to indicate how much you agree with each item below

| Items | strongly disagree | disagree | neither agree nor disagree | agree | strongly agree |
| --- | --- | --- | --- | --- | --- |
| 1. When I receive a poor grade, I usually feel that the main reason is that I haven't studied enough for that course | 1 | 2 | 3 | 4 | 5 |
| 2. My enjoyment of a social occasion is almost entirely dependent on the personalities of the other people who are there | 1 | 2 | 3 | 4 | 5 |
| 3. If I were to receive low marks it would cause me to question my academic ability | 1 | 2 | 3 | 4 | 5 |
| 4. Making friends is a funny business; sometime I have to chalk up my successes to luck | 1 | 2 | 3 | 4 | 5 |
| 5. If I did not get along with others, it would tell me that I hadn't put much effort into the pursuit of social goals | 1 | 2 | 3 | 4 | 5 |
| 6. Some of the times that I have gotten a good grade in a course, it was due to the teacher's easy grading scheme | 1 | 2 | 3 | 4 | 5 |
| 7. It seems to me that failure to have people like me would show my ignorance in inter-personal relationships | 1 | 2 | 3 | 4 | 5 |
| 8. Sometimes my success on exams depends on some luck | 1 | 2 | 3 | 4 | 5 |
| 9. In my case, the good grades I receive are always the direct result of my efforts. | 1 | 2 | 3 | 4 | 5 |
| 10. No matter what I do, some people just don't like me | 1 | 2 | 3 | 4 | 5 |
| 11. The most important ingredient in getting good grades is my academic ability. | 1 | 2 | 3 | 4 | 5 |
| 12. Often chance events can play a large part in causing rifts between friend | 1 | 2 | 3 | 4 | 5 |
| 13. Maintaining friendships requires real effort to make them work | 1 | 2 | 3 | 4 | 5 |
| 14. In my experience, once a professor gets the idea you're a poor student, your work is much more likely to receive poor grades than if someone else handed it in | 1 | 2 | 3 | 4 | 5 |
| 15. It seems to me that getting along with people is a skill | 1 | 2 | 3 | 4 | 5 |
| 16. Some of my lower grades have seemed to be partially due to bad breaks | 1 | 2 | 3 | 4 | 5 |
| 17. When I fail to do as well as expected in school, it is often due to a lack of effort on my part | 1 | 2 | 3 | 4 | 5 |
| 18. Some people can make me have a good time even when I don't feel sociable | 1 | 2 | 3 | 4 | 5 |
| 19. If I were to fail a course it would probably be because I lacked skill in that area | 1 | 2 | 3 | 4 | 5 |
| 20. In my experience, making friends is largely a matter of having the right breaks | 1 | 2 | 3 | 4 | 5 |
| 21. When I hear of a divorce, I suspect that the couple probably did not try enough to make their marriage work | 1 | 2 | 3 | 4 | 5 |
| 22. Some of my good grades may simply reflect that these were easier courses than most | 1 | 2 | 3 | 4 | 5 |
| 23. I feel that people who are often lonely are lacking in social competence | 1 | 2 | 3 | 4 | 5 |
| 24. I feel that some Of my good grades depend to a considerable extent on chance factors, such as having the right questions show up on an exam | 1 | 2 | 3 | 4 | 5 |
| 26. Some people just seem predisposed to dislike me | 1 | 2 | 3 | 4 | 5 |
| 27. I feel that my good grades reflect directly on my academic ability | 1 | 2 | 3 | 4 | 5 |
| 28. I find that the absence of friendships is often a matter of not being lucky enough to meet the right people | 1 | 2 | 3 | 4 | 5 |
| 29. In my case, success at making friends depends on how hard I work at it | 1 | 2 | 3 | 4 | 5 |
| 30. Often my poorer grades are obtained in courses that the professor has failed to make interesting | 1 | 2 | 3 | 4 | 5 |
| 31. Having good friends is simply a matter of one's social skill | 1 | 2 | 3 | 4 | 5 |
| 32. My academic low points sometimes make me think I was just unlucky | 1 | 2 | 3 | 4 | 5 |
| 33. Poor grades inform me that I haven't worked hard enough | 1 | 2 | 3 | 4 | 5 |
| 34. To enjoy myself at a party I have to be surrounded by others who know how to have a good time | 1 | 2 | 3 | 4 | 5 |
| 35. If I were to get poor grades I would assume thatI lacked ability to succeed in those courses. | 1 | 2 | 3 | 4 | 5 |
| 36. If my marriage were a long, happy one, I'd say that I must just be very lucky | 1 | 2 | 3 | 4 | 5 |
| 37. In my experience, loneliness comes from not trying to be friendly | 1 | 2 | 3 | 4 | 5 |
| 38. Sometimes I get good grades only because the course material was easy to learn | 1 | 2 | 3 | 4 | 5 |
| 39. In my experience, there is a direct connection between the absence of friendship and being socially inept | 1 | 2 | 3 | 4 | 5 |
| 40. Sometimes I feel that I have to consider myself lucky for the good grades I get | 1 | 2 | 3 | 4 | 5 |
| 41. I can overcome all obstacles in the path of academic success if I work hard enough | 1 | 2 | 3 | 4 | 5 |
| 42. It is almost impossible to figure out how I have displeased some people | 1 | 2 | 3 | 4 | 5 |
| 43. When I get good grades, it is because of my academic competence | 1 | 2 | 3 | 4 | 5 |
| 44. Difficulties with my friends often start with chance remarks | 1 | 2 | 3 | 4 | 5 |
| 45. If my marriage were to succeed, it would have to be because I worked at it | 1 | 2 | 3 | 4 | 5 |
| 46. Some low grades I've received seem to me to reflect the fact that some teachers are just stingy with marks | 1 | 2 | 3 | 4 | 5 |
| 47. It is impossible for me to maintain close relations with people without my tact and patience | 1 | 2 | 3 | 4 | 5 |
| 48. Some of my bad grades may have been a function of bad luck, being in the wrong course at the wrong time | 1 | 2 | 3 | 4 | 5 |

Diener, E., Emmons, R. A., Larsen, R. J., & Griffin, S. (1985). The Satisfaction With Life Scale. *J Pers Assess, 49*(1), 71-75. doi:10.1207/s15327752jpa4901_13

Lefcourt, H. M., Von Baeyer, C. L., Ware, E. E., & Cox, D. J. (1979). The multidimensional-multiattributional causality scale: The development of a goal specific locus of control scale. *Canadian Journal of Behavioural Science/revue Canadienne Des Sciences Du Comportement, 11*(4), 286-304.

Watson, D., Clark, L. A., & Tellegen, A. (1988). Development and validation of brief measures of positive and negative affect: the PANAS scales. *J Pers Soc Psychol, 54*(6), 1063-1070. doi:10.1037//0022-3514.54.6.1063
