# Supplementary material for: Impact of OXTR Polymorphisms on Subjective Well-Being: The Intermediary Role of Attributional Style
Source: Front Genet. 2022 Feb 9;12:763628. doi: 10.3389/fgene.2021.763628 (PMC8864163; doi:10.3389/fgene.2021.763628)
Supplement: Supplementary file 3 [file DataSheet1.DOCX]

**Supplementary**

1. The description of SAWL and PANAS used in the calculation of SWB

Life satisfaction were assessed with the Satisfaction With Life Scale (SWLS) (E. Diener, Emmons, Larsen, & Griffin, 1985). The SWLS score is the sum of 5 item scores; the scale range is from 5 (lowest satisfaction) to 35 (highest satisfaction) (Appendix a). The reliability coefficient (Cronbach’s alpha) of the scale in this study is 0.88, consistent with the value reported in original research on Chinese sample (Bai, Wu, Zheng, & Ren, 2011). The mean score for satisfaction with life (Mean ± SD: 20.35±5.89) was also similar to the previous report (Mean ± SD: 20.32±5.99) (Bai et al., 2011).

Positive affect and negative affect were measured with the Positive Affect (PA) and Negative Affect (NA) subscales from the Positive and Negative Affect Scale (PANAS) (Watson, Clark, & Tellegen, 1988). The PANAS consists of a Positive affect scale (10 items) and a Negative affect scale (10 items) (Appendix b). Each scale (PA and NA) was a Likert-type scale ranging from very slightly or not at all (1) to extremely (5) indicating the extent to which respondents usually feel the words that describe a series of feelings and emotions. Total scores on each scale (PA and NA) are obtained by adding the scores for each item. The Cronbach’s alpha varied from 0.86 to 0.90 for the PA scale and from 0.84 to 0.87 for the NA scale (Watson et al., 1988). In the present sample, the Cronbach’s α of the PA scale was 0.87 and of NA scale was 0.92. The final score of SWB was calculated by confirmatory factor analyses (CFA) on these three factors (Bentler & M, 1980). The result of the CFA provided an excellent fit to the observed data [χ^2^(3) = 426.475, p<0.001]. The loadings of the measured variables on the latent variable of SWB were statistically significant at the 0.001 level, which implied that SWB has been adequately measured by its respective indicators.

1. The mediating effect of the attributional styles on the association between *OXTR* rs2254298 and SWB after controlling the effect of gender. Together with what is described in the main body of the article, we have constructed four models. The detailed structural equation models are shown in Figure 1,2,3. We tested whether the fitted index of the constructed structural equation model conformed to the requirements (The model fit is considered to be accepted by convention if the RMSEA is less than or equal to 0.08 and the CFI and TLI should be equal to or greater than 0.90). After comparing these models, we selected the optimal model: the path from luck to context attribution mediates the association between OXTR and SWB.

(A) The mediating effect of Ability attribution, Luck attribution Context attribution in a parallel model. The results are shown in the Table 1 and Figure 1 below. Model fit: χ^2^_(11)_ = 570.696, TLI = -0.048, CFI = 0.451, RMSEA = 0.285, and SRMR = 0.187 (Number of Bootstrap Resample: 1000). The model fit was not good.

**Table 1 Multiple mediation model test with bootstrapping.**

| **Mediators** | **Estimate** | **SE** | **z-value** | **95%*CI*** | ***P* value** |
| --- | --- | --- | --- | --- | --- |
| Direct Effect | 0.089 | 0.153 | 2.015 | (0.003, 0.175) | **0.042** |
| Indirect Effect 1 _(O→A→S)_ | -0.007 | 0.006 | -1.184 | (-0.020, 0.005) | 0.237 |
| Indirect Effect 2 _(O→L→S)_ | 0.010 | 0.009 | 1.166 | (-0.007, 0.027) | 0.244 |
| Indirect Effect 3 _(O→C→S)_ | 0.014 | 0.007 | 1.951 | (-0.000, 0.028) | 0.051 |
| Total Indirect Effect | 0.017 | 0.010 | 1.617 | (-0.004, 0.037) | **0.106** |
| Total Effect | -0.072 | 0.067 | -1.082 | (-0.204, 0.059) | **0.279** |


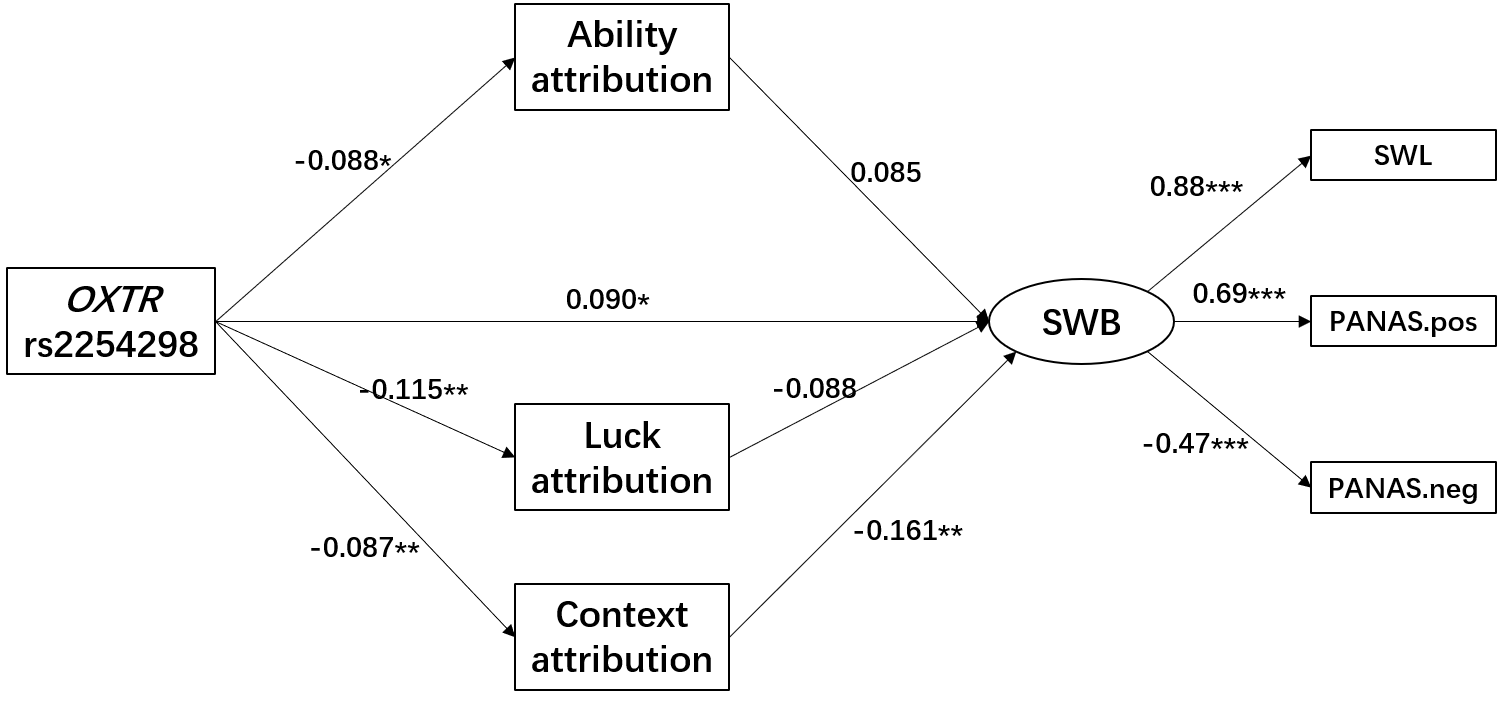


**Figure 1 Model A**

(B) The mediating effect of Luck attributional style and Context attribution style in a parallel model.

The results show that the fit indexes are χ^2^_(7)_ = 314.32, TLI = 0.135, CFI = 0.596, RMSEA = 0.265, and SRMR = 0.141 (Number of Bootstrap Resample: 1000). Figure 2 depicts the mediating effect model. As a result, the model fit was not good. Thus, this model was not taken into consideration.


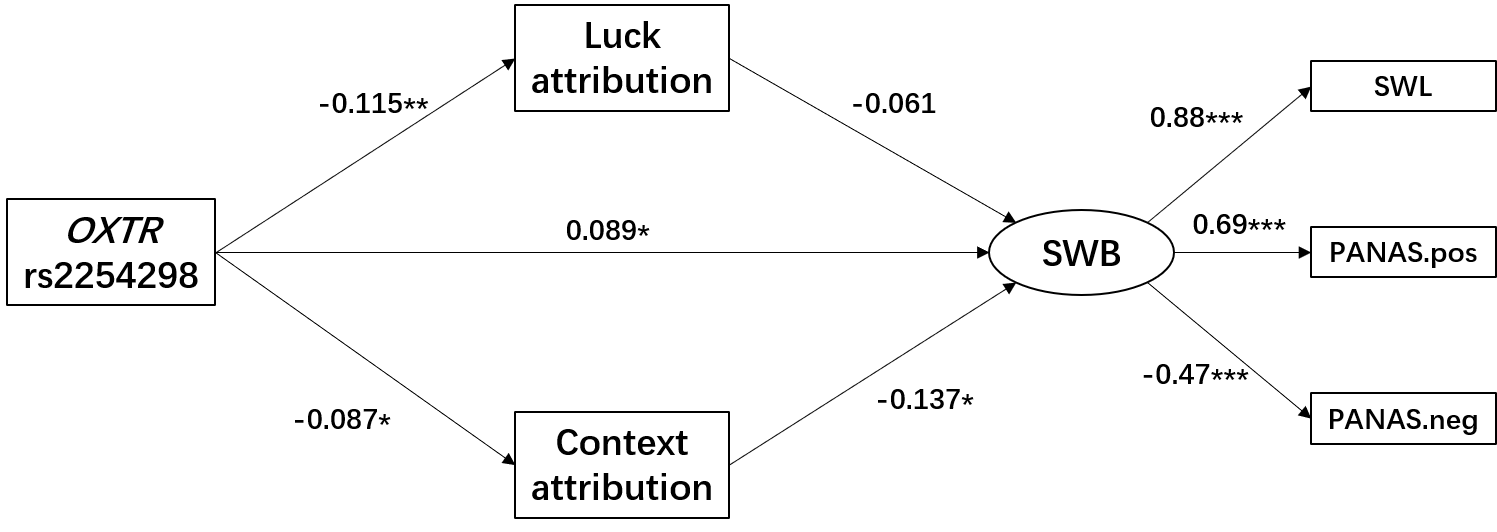


**Figure 2 Model B**

(C) Chain mediating path: from context attribution to luck attribution

We checked on the model fit of the model depicted in Figure 3 below. Model fit: χ^2^_(6)_ = 37.515, TLI = 0.897, CFI = 0.959, RMSEA = 0.092, and SRMR = 0.045 (Number of Bootstrap Resample: 1000). This model showed inadequate fit because the RMSEA was greater than 0.08 and TLI was less than 0.90.


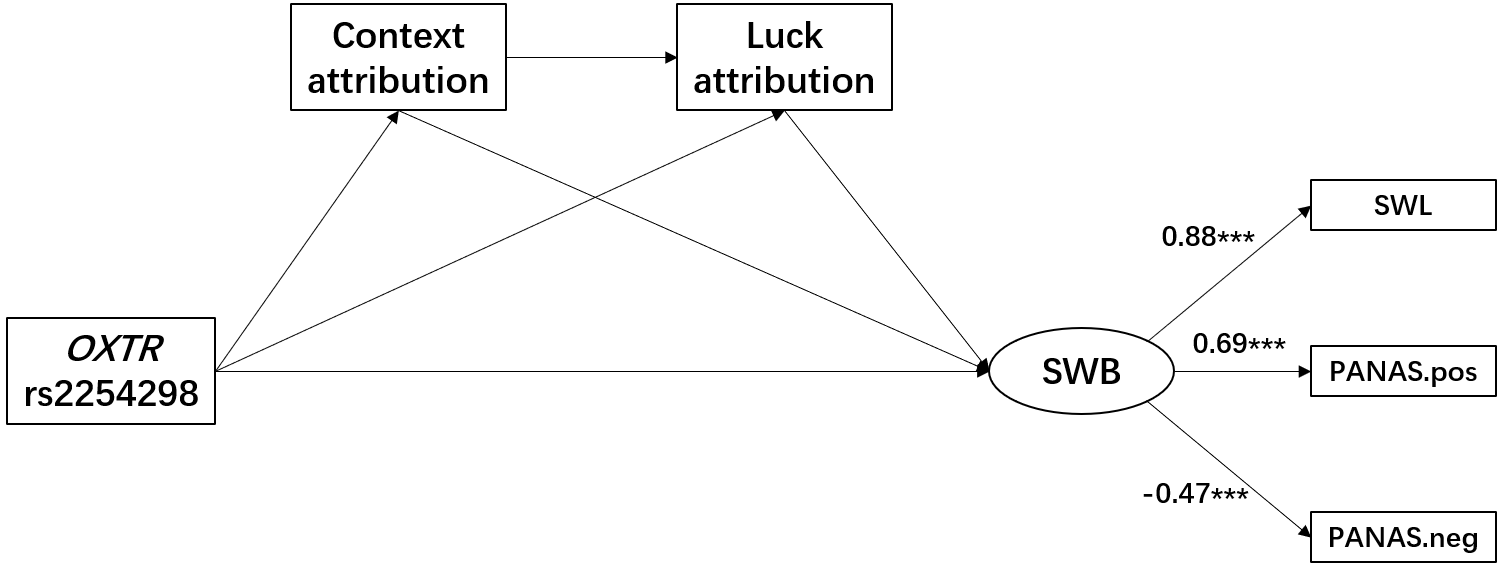


**Figure 3 Model C**

1. Results that exclude non-Han Chinese and individuals with a family history of psychosis (N=569)

After excluding non-Han Chinese and individuals with a family history of psychosis, the final sample size was 569, which did not achieve statistical validity based on power analysis (requirement: 592-892). Therefore, this result may not be credible. The model showed a less adequate fit: χ^2^(6) = 36.645, RMSEA = 0.095, CFI = 0.956, TLI = 0.891, with bootstrapping the mediating effect 1000 times (Figure 4). Table 2 present the standardized path coefficients for the effects of *OXTR* rs2254298 on SWB in the mediation model.

**Table 2 Multiple mediation model test with bootstrapping**

| **Mediators** | **Estimate** | **SE** | **z-value** | **95%*CI*** | ***P* value** |
| --- | --- | --- | --- | --- | --- |
| Direct Effect | 0.077 | 0.043 | 1.782 | (-0.008, 0.162) | 0.072 |
| Indirect Effect 1 _(O→L→S)_ | 0.007 | 0.010 | 0.696 | (-0.012, 0.025) | 0.486 |
| Indirect Effect 2 _(O→C→S)_ | 0.003 | 0.004 | 0.734 | (-0.005, 0.011) | 0.463 |
| Indirect Effect 3 _(O→L→C→S)_ | 0.011 | 0.005 | 2.029 | (0.000, 0.022) | **0.042** |
| Total Indirect Effect | 0.021 | 0.010 | 2.068 | (0.001, 0.040) | **0.039** |
| Total Effect | 0.098 | 0.045 | 2.166 | (0.009, 0.186) | **0.030** |

Note. 95%*CI*: 95% Confidence Interval.

Number of Bootstrap Resample: 1000.


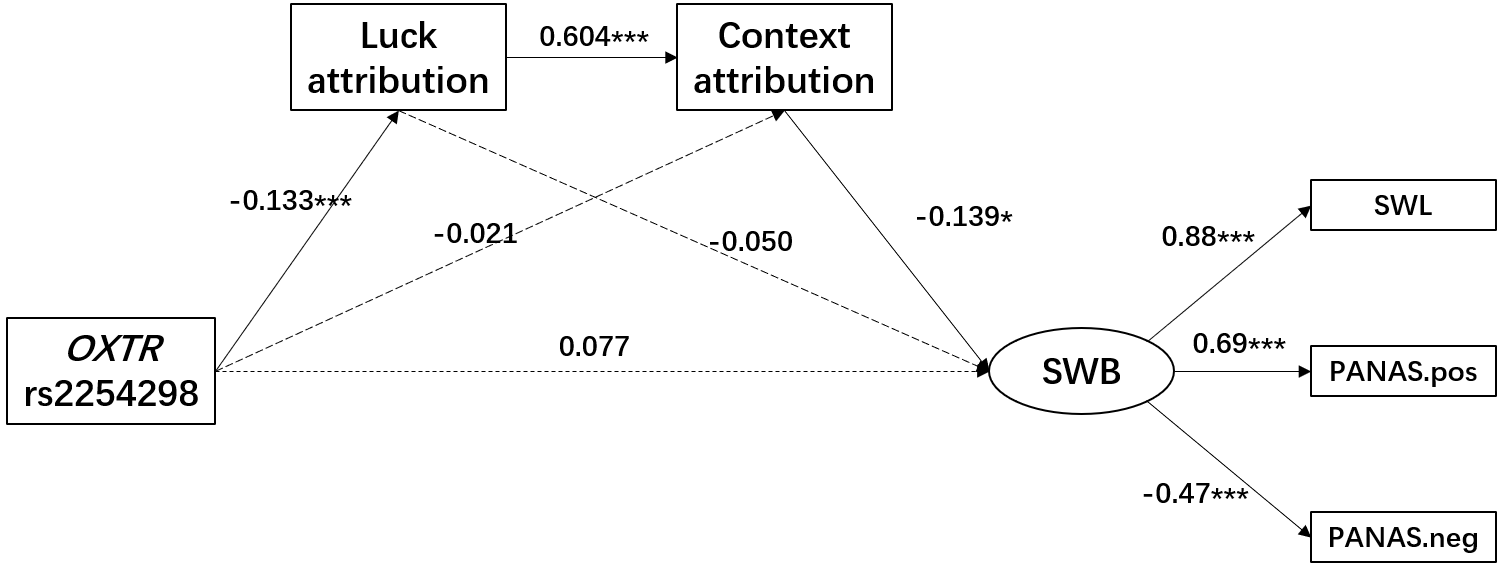


**Figure 4**
